# Anterior Chamber Angle Shape Analysis and Classification of Glaucoma in SS-OCT Images

**DOI:** 10.1155/2014/942367

**Published:** 2014-08-05

**Authors:** Soe Ni Ni, J. Tian, Pina Marziliano, Hong-Tym Wong

**Affiliations:** ^1^School of Electrical and Electronic Engineering, Nanyang Technological University, 50 Nanyang Avenue, Singapore 639798; ^2^Department of Ophthalmology, Tan Tock Seng Hospital, 11 Jalan Tan Tock Seng, Singapore 308433; ^3^National Healthcare Group Eye Institute, Singapore

## Abstract

Optical coherence tomography is a high resolution, rapid, and noninvasive diagnostic tool for angle closure glaucoma. In this paper, we present a new strategy for the classification of the angle closure glaucoma using morphological shape analysis of the iridocorneal angle. The angle structure configuration is quantified by the following six features: (1) mean of the continuous measurement of the angle opening distance; (2) area of the trapezoidal profile of the iridocorneal angle centered at Schwalbe's line; (3) mean of the iris curvature from the extracted iris image; (4) complex shape descriptor, fractal dimension, to quantify the complexity, or changes of iridocorneal angle; (5) ellipticity moment shape descriptor; and (6) triangularity moment shape descriptor. Then, the fuzzy *k* nearest neighbor (fkNN) classifier is utilized for classification of angle closure glaucoma. Two hundred and sixty-four swept source optical coherence tomography (SS-OCT) images from 148 patients were analyzed in this study. From the experimental results, the fkNN reveals the best classification accuracy (99.11 ± 0.76%) and AUC (0.98 ± 0.012) with the combination of fractal dimension and biometric parameters. It showed that the proposed approach has promising potential to become a computer aided diagnostic tool for angle closure glaucoma (ACG) disease.

## 1. Introduction

The detection of the angle closure glaucoma is important for preventing irreversible blindness. Since vision loss from glaucoma cannot be recovered, improved screening and detection methods for the angle closure glaucoma are essential to preserve vision and maintain a good quality of life. Although glaucoma commonly progresses to blindness over many years, acute angle closure can result in permanent blindness in a matter of hours. Studies suggest that glaucomatous optic neuropathology can be prevented when effective prophylactic treatment such as laser peripheral iridotomy is performed at an early and appropriate time for the eyes with anatomically narrow angles [1]. Angle closure glaucoma is characterized by obstruction of aqueous fluid drainage through the trabecular meshwork from the eye's anterior chamber. The width of the angle is one factor affecting the drainage of aqueous humor. A wide unobstructed iridocorneal angle allows sufficient drainage of aqueous humor, whereas a narrow angle may impede the drainage system and leave the patient susceptible to angle closure glaucoma.

The imaging of angle between the iris and the cornea is the key for open angle and closed angle glaucoma diagnosis. Early detection of ACG using imaging technology has recently gained much clinical interest. Gonioscopy [2], is considered the gold standard technique to examine the iridocorneal angle, but it is subjective and dependent on the operator. OCT is a high resolution, rapid, and noninvasive screening tool for angle closure glaucoma and its technology has evolved rapidly from time-domain to spectral-domain. Swept source OCT (Casia SS-1000) [3] is a newly developed novel imaging technology which provides a detailed examination of the structures of anterior chamber (AC). It is a Fourier-domain system and is designed specifically for imaging the anterior segment. With a substantial improvement in scan speed (30,000 A-scans per second), the anterior chamber angles can be imaged in 128 cross-sections (each with 512 A-scans) 360 degree around the anterior segment in 1.2 seconds. High-definition SS-OCT (SS-OCT) imaging has the potential to become an important tool for the assessment of the anterior chamber angle and detection of angle closure [4, 5].

Anterior segment SS-OCT imaging has significantly altered the diagnosis and evaluation of ACG. The information gained with new imaging modalities provides clinicians with both qualitative and quantitative information about anatomical structure of the anterior chamber. Hu et al. [6] compared gonioscopy with Visante and Cirrus optical coherence tomography (OCT) for identifying angle structures and the presence of angle closure in patients with glaucoma. Several techniques to assess the anterior chamber are based on manual or automatic detection of some landmarks such as scleral spur (SS) [7] and Schwalbe's line (SL) [8, 9]. However, the existing methods use a single distance or area, for example, AOD500, TISA500, and AOD_sl_, for measurement of iridocorneal angle, without consideration of the whole angle profile. Cheung et al. [10] showed that an irregular iris surface made the AOD_sl_ very inaccurate. Our previous study [11] measured two new parameters, mAOD and AT_sl_, based on the continuous AOD to assess the anterior chamber angle. These two new parameters tried to overcome the limitations in the single measurement of AOD when the iris surface is irregular and the angle is more occludable [10].

In addition to these two parameters, the iris curvature must also be considered. The iris curvature of anterior segment OCT (AS-OCT) is usually calculated by drawing a line from the most peripheral to the most central points of the iris pigment epithelium [12], and then a perpendicular line is extended from this line to the iris pigment epithelium at the point of the greatest convexity. However, the iris pigment epithelium cannot be exactly detected, only the stoma. The segmentation of the iris lower boundary is challenging due to the low contrast in this region. The nature of the angle dynamics which has not been considered yet in the previous studies, still involved in different forms of angle closure or open angle glaucoma. Hence, we propose to measure the iris curvature denoted by *H*_iris_ from the extracted iris image.

Besides, we assume that the iridocorneal angle is the irregular shape and the complexity or changes of the iridocorneal angle can be measured quantitatively and qualitatively with shape descriptors. The shape analysis might give more freedom in computation and is less sensitive to the accurate detection of the landmarks—the scleral spur (SS) and Schwalbe's line (SL). Using this approach, the shape analysis may be successfully applied both quantitatively and objectively to characterize angle shape of anterior chamber of SS-OCT images. An important property of shapes is their complexity. The complex and erratic shape description in terms of self-similarity was introduced by Mandelbrot [13]. The concept of fractal dimension (FD) is useful in the measurement, analysis, and classification of shape and texture. The fractal dimension, therefore, might serve as a sensitive descriptor of the iridocorneal angle shape. Fractals provide a new field for characterization of irregularity and complexity, yet self-similar structures in nature.

However, although FD has been used extensively in characterizing self-affinity in various kinds of biomedical research [13, 14], little attention has been paid in automated glaucoma subtype classification using the feasibility of fractal and multifractal theory on retinal nerve fiber layer (RNFL) and optic disc [15]. Another approach in shape analysis is founded on convexity [16]. The measurement of moment invariants like the triangularity (*T*_*I*_) and ellipticity (*E*_*I*_) [17] was successfully used in several applications like the classification of mammographic masses and lung field boundaries [18]. Both shape analyses are a translation, rotation, and scale of the object and general affine transformations [19]. Therefore, the paper proposes a new strategy to analyze the iridocorneal angle by morphological shape analysis as well as biometric angle parameters.

In this paper, we present an automatic angle closure glaucoma detection system based on machine learning and image analysis for the estimation of quantitative parameters to classify SS-OCT images into two classes: open angle and angle closure glaucoma. We propose new strategies of feature extraction methods: (1) based on measurement of biometric parameters and (2) based on the shape analysis of iridocorneal angle to capture much more information.

The rest of the paper is organized as follows.  Section 2 describes the overview of the algorithm for preprocessing, feature extractions, and classification method of angle closure glaucoma. The experiment and results are presented in Section 3. Finally Section 4 concludes the paper with the future work.

## 2. Methods

**Figure 1:**
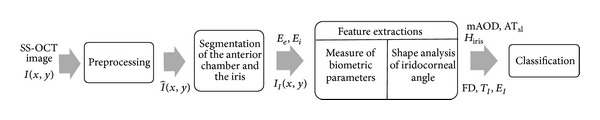
Overview of the automatic classification system for open angle and angle closure glaucoma using SS-OCT images.

**Figure 2:**
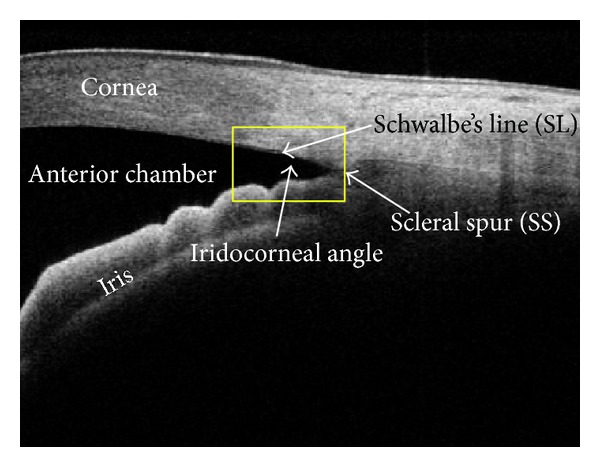
Anterior chamber imaging showing Schwalbe's line and yellow bounding box for region of interest (ROI) for angle analysis.

The architecture of the overall proposed system is shown in Figure 1. It mainly consists of four steps: preprocessing, segmentation of anterior chamber, anterior chamber angle analysis for feature extractions, and classification of angle closure and open angle glaucoma. This section starts by defining the notation that will be used throughout the paper and Figure 2 shows the region of interest for iridocorneal angle analysis.

Firstly, the original image denoted by *I*(*x*, *y*) is preprocessed to remove the vertical saturation artifacts as in [11] and the processed image is denoted by $\hat{I}(x,y)$, where (*x*, *y*)∈[1, *M*]×[1, *N*] are the pixels coordinates and *M* × *N* is the size of the image; then $\hat{I}(x,y)$ is segmented to detect the cornea, the iris, and the anterior chamber. The lower boundaries of the cornea *E*_*e*_ and inner boundary of the iris *E*_*i*_ are detected using the same approach as the method in [9], and the iris image denoted by *I*_*I*_(*x*, *y*) is extracted. The six features are extracted to quantify the anterior chamber; the anterior chamber assessment parameters are mAOD, AT_sl_, the mean curvature of the iris *H*_iris_, and shape descriptors FD, *T*_*I*_, and *E*_*I*_. Lastly, the classification step is performed. The short descriptions of each step are presented in the following subsequent sections. The extended description of each step can be found in the appendices.

### 2.1. Notation

The definitions of the variables used in this paper are listed here:

1. original gray scale image: *I*(*x*, *y*), where (*x*, *y*)∈[1, *M*]×[1, *N*];
2. the preprocessed image: $\hat{I}(x,y)$, where (*x*, *y*)∈[1, *M*]×[1, *N*];
3. the segmented corneal endothelium, the anterior surface of the iris, and anterior chamber mask in *I*(*x*, *y*): *E*_*e*_, *E*_*i*_, and *M*_AC⁡_;
4. the extracted iris image: *I*_*I*_(*x*, *y*);
5. automatically detected Schwalbe's line: SL;
6. angle opening distance: AOD;
7. mean of the continuous measurement of the angle opening distance: mAOD;
8. area of the trapezoidal profile of the iridocorneal angle centered at Schwalbe's line: AT_sl_;
9. mean of the iris curvature from the extracted iris image; *H*_iris_;
10. the extracted iridocorneal angle image: *I*_IRA_(*x*, *y*), where $\left(x,y)\in [1,\bar{M}]\times [1,\bar{N}\right]$;
11. complex shape descriptor to quantify the complexity or changes of iridocorneal angle: fractal dimension (FD);
12. ellipticity moment shape descriptor: *E*_*I*_;
13. triangularity moment shape descriptor: *T*_*I*_.

### 2.2. Preprocessing of SS-OCT Images

**Figure 3:**
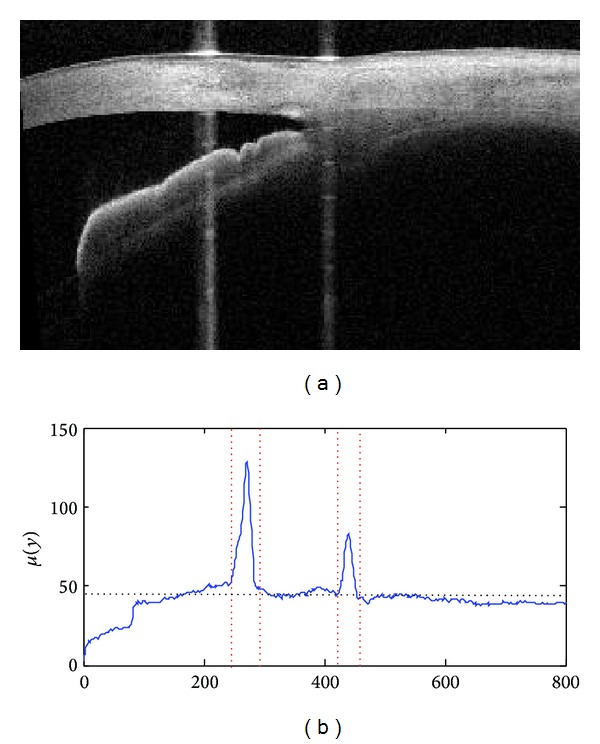
(a) The original closed angle SS-OCT image with contaminated vertical saturation artifact; (b) detection of the artifact by searching the abrupt changes in intensities of the SS-OCT images where the vertical dotted lines denote the region of detected vertical artifact and the horizontal dotted lines denote the mean intensity of the SS-OCT images.

It is observed that some SS-OCT images contain vertical saturation artifacts, which hinder the accurate interpretation of the image if thresholding and component labeling are used for the segmentation. Hence, reducing and removing the artifacts are performed prior to the segmentation step. As illustrated in Figure 3(a), the vertical saturation artifacts are marked by sudden increases in intensity as compared to surrounding area and are caused by the saturation of intensity due to the strong reflection in certain locations. Therefore, we detected such artifacts by searching for abrupt changes in the average intensity of each column as shown in Figure 3(b).

### 2.3. Segmentation of Anterior Chamber and Iris

After removing the vertical saturation artifacts, the segmentation was performed in the SS-OCT images. The method consists of segmenting the anterior chamber, the cornea, and the iris and extracting their edges. The anterior chamber region, the lower cornea boundary, and the upper iris boundary are extracted by image segmentation method. The details of segmentation can be found in appendices.

### 2.4. Feature Extractions

This section describes the analysis of ACA for the extraction of six features by the biometric parameter measurement and shape analysis.

#### 2.4.1. Measurement of Biometric Parameters

The biometric parameters, previously proposed in [11], from the iridocorneal angle based on the continuous measurement of AOD are quantified as follows.

**Figure 4:**
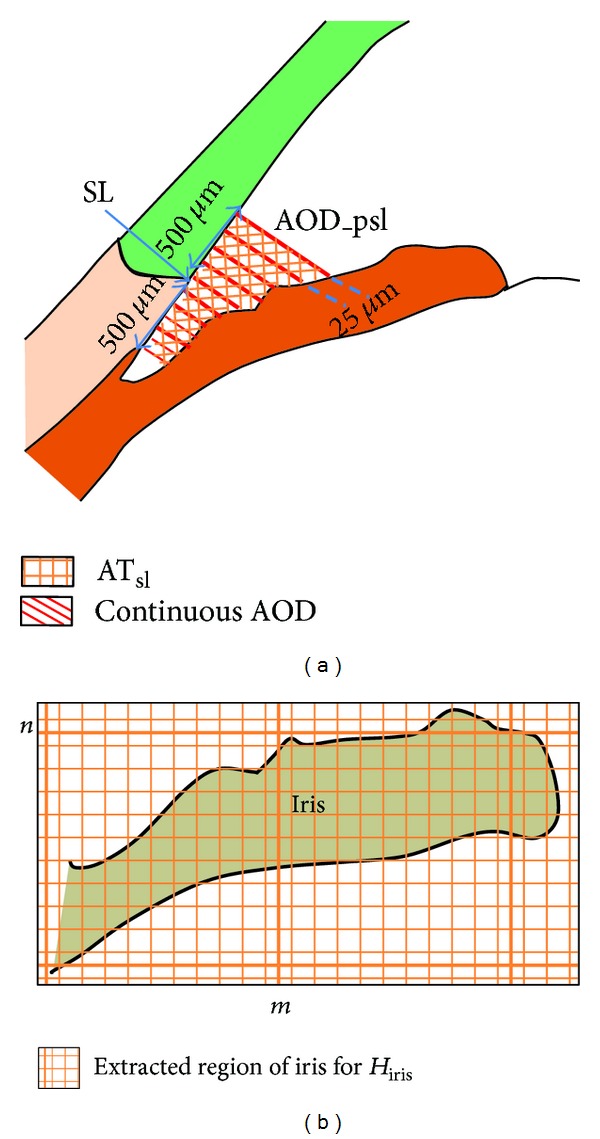
(a) Biometric parameter measurement in SS-OCT image. (b) Extracted region of iris to compute iris curvature. SL: Schwalbe's line, AT_sl_: area of trapezoidal profile of iridocorneal angle centered at SL, AOD_psl: angle opening distance 500 *μ*m posterior from SL, *H*_iris_: the mean curvature of iris.

- mAOD: the average of continuous serial AOD measured every 25 *μ*m away from the SL till 500 *μ*m in both directions (anterior and posterior to/from Schwalbe's line) as shown in Figure 4(a).
- AT_sl_: the trapezoidal area of iridocorneal angle bounded by the angle recess, AOD_psl line, corneal endothelium, and anterior surface of the iris as shown in Figure 4(a).
- *H*_iris_: the mean iris curvature measured from the whole iris image as shown in Figure 4(b).

#### 2.4.2. Anterior Chamber Shape Analysis

**Figure 5:**
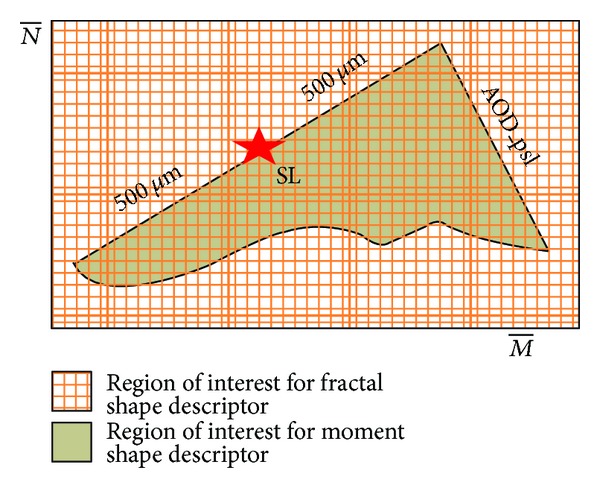
Shape analysis on the region of interest in SS-OCT image where SL is Schwalbe's line and AOD_psl is the angle opening distance 500 *μ*m posterior from SL.

After calculating the biometric parameters, we performed anterior chamber shape analysis on the selected region of iridocorneal angle image as shown in Figure 5. We utilized the fractal complex shape descriptor on the iridocorneal angle image denoted by *I*_IRA_(*x*, *y*), where $\left(x,y)\in [1,\bar{M}]\times [1,\bar{N}\right]$ are the pixels coordinates and $\bar{M}\times \bar{N}$ is the size of the extracted angle image. Moment shape descriptors such as the triangularity and ellipticity are used to compare the performance of fractal dimension analysis. The angle structure configuration is then quantified by the following three features:

1. complex shape descriptor to quantify the complexity or changes of iridocorneal angle, fractal dimension (FD);
2. ellipticity moment shape descriptor (*E*_*I*_);
3. triangularity moment shape descriptor (*T*_*I*_).

### 2.5. Classification

The ability of each feature extraction method to separate open angle and angle closure glaucoma is quantified by the fuzzy* k*-nearest neighbour classifier [20]. The basis of the fuzzy k-NN algorithm is to assign membership as a function of the vector's distance from its* k*-nearest neighbors and those neighbors' memberships in the possible classes.

## 3. Experiments and Results

The Singaporean Chinese population study by the Singapore National Eye Centre recruited 148 subjects (90 females and 45 males) with average age of 59.48 ± 8.97. All subjects underwent a standard examination of dark room gonioscopy and the anterior segment imaging by SS-OCT on the same day. The Casia SS-1000 OCT (Tomey, Nagoya, Japan) was used as the imaging modality to visualize the anterior segment of the eye. We used the 2D angle high-definition (HD) mode of SS-OCT imaging with the (8 mm, 8 mm) scan dimension. In the HD scan mode, the identification of both the SL and SS was possible in over 98% of SS-OCT images [21]. The ACA was graded using the modified Shaffer grading system in each SS-OCT image. The doctor examined four different quadrants of the eye, namely, inferior, superior, nasal, and temporal (I, S, N, and T) scanning. A subset of 29 subjects (23.4%) was imaged bilaterally to assess the differences between eyes.

**Figure 6:**
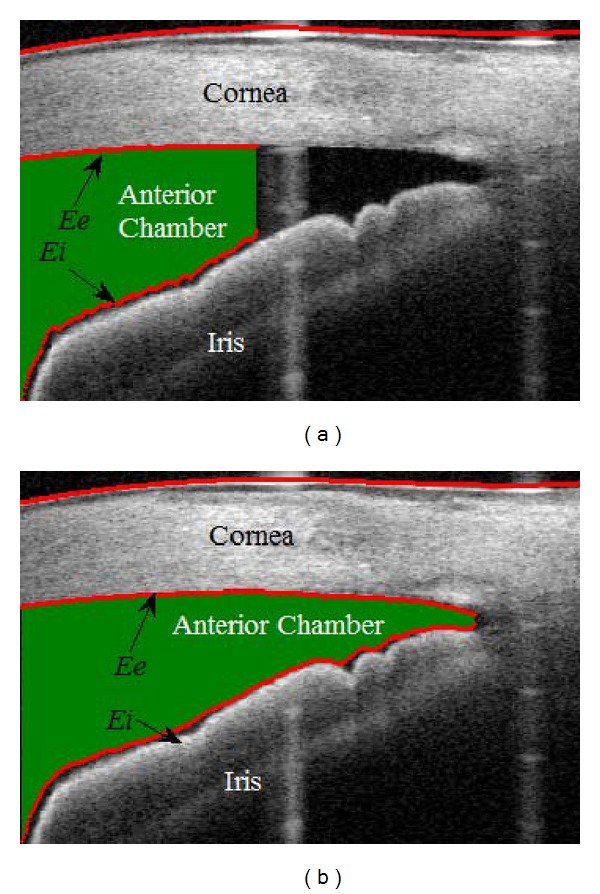
(a) Segmentation of anterior chamber without artifact removal; (b) segmentation of anterior chamber, the cornea, and the anterior of iris surface with artifact removal in SS-OCT image. Green region is extracted anterior chamber and red lines are edges of cornea and anterior of iris.

We selected the images with two criteria: (1) SL could be identified automatically and (2) only one image from the four scans of each eye, for less bias for classifier. So, in total 264 SS-OCT HD images in which SL could be seen were selected from the dataset for further analysis. They were 132 nasal scan images, 70 temporal scan images, 29 superior scan images, and 33 inferior scan images. Using gonioscopic Shaffer grading as the gold standard, 135 images with closed angle (1.131 ± 0.72 grade) and 129 images with open angle (3.321 ± 0.469 grade) were evaluated for the analysis. The vertical saturation artifacts were presented in 77 images (~46%) out of 264 images in the dataset on various regions of image. So, it is necessary to perform artifact removal for affected images prior to image segmentation.  Figure 6 shows the segmentation results of with and without artifact removal in SS-OCT image. From the results as shown in Figure 6, we found that the preprocessing steps relatively improved the segmentation of the anterior chamber region.

**Figure 7:**
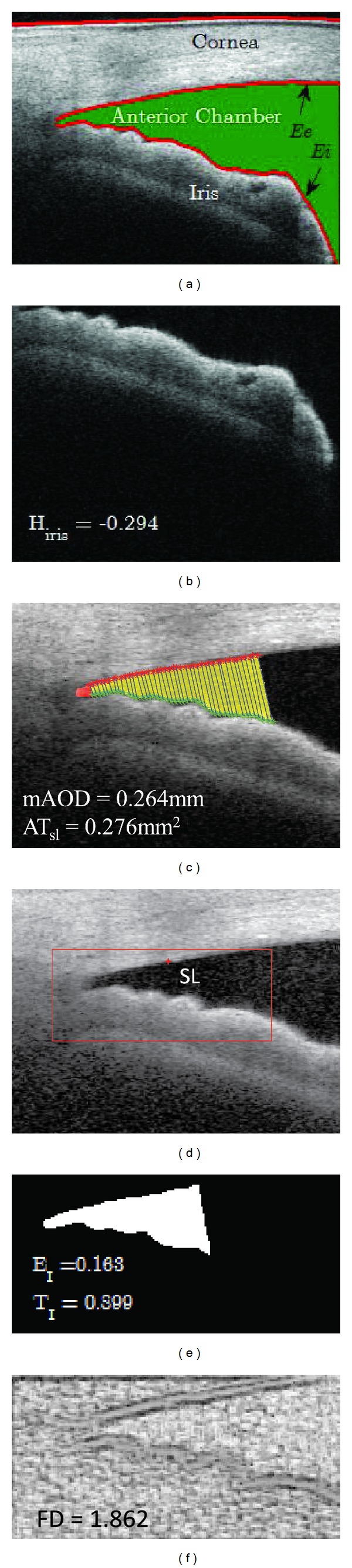
The results of segmentation and feature extractions on SS-OCT image; (a) segmentation of anterior chamber; (b) extracted iris image for curvature analysis; (c) the resulting angle profile for biometric parameter measurement; (d) region of interest for fractal dimension analysis; (e) extracted shape for moment shape analysis; and (f) fractal image of extracted region of SS-OCT image.

Figure 7(a) shows the segmentation results of the anterior chamber, the upper cornea, the lower cornea, and the inner boundary of the iris. The extracted iris image is shown in Figure 7(b) which yields the iris curvature of −0.294.  Figure 7(c) shows an example of the resulting angle profile consisting of 40 continuous AOD centered on SL which reveals 0.262 mm for mAOD and 0.276 mm^2^ for AT_sl_, respectively. Then, we find the region of interest for the fractal dimension and the moment shape analysis as shown in Figure 7(d). The results of the triangularity and the ellipticity moment shape analysis are 0.899 and 0.165, respectively, as shown in Figure 7(e).  Figure 7(f) shows the fractal image of the region of interest of Figure 7(d) and its estimated average FD is 1.862.

**Figure 8:**
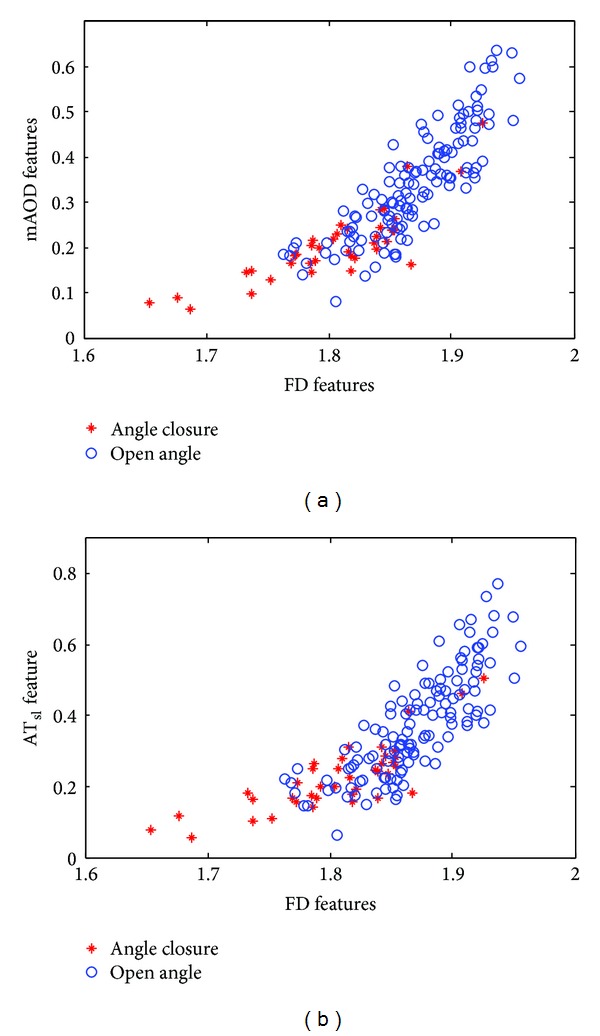
(a) Scatter plot of FD features versus mAOD for 264 images and (b) scatter plot of FD features versus AT_sl_ for 264 images.

The comparison of the iridocorneal angle features between open angle and angle closure glaucoma SS-OCT images using gonioscopic grading as a reference is illustrated in Table 1. The fractal dimensions of open and closed angle are 1.87 and 1.84, respectively, as shown in Table 1. The results of fractal dimension depend slightly on the specific images of open angle and angle closure glaucoma. All the features of closed angle SS-OCT images are smaller than those of the open angle SS-OCT images. The coefficients of variance (COVs) of FD are smaller in both open and closed angle images. The COVs of *H*_iris_ are the largest in both open and closed angle SS-OCT images. So, *H*_iris_ results are considered to be high variance and do not necessarily correspond to closed and open angle images in this study.  Figure 8 shows the scatter plot of FD, mAOD, and AT_sl_ features for both open angle and closed angle SS-OCT images. After the feature vectors were computed, training, cross-validation, and testing sets were formed by 1476 vectors (264 images × 6 features): 3 biometric features (mAOD, AT_sl_, and *H*_iris_) and 3 shape descriptors (FD, *T*_*I*_, and *E*_*I*_).

Then, we perform the feature selections from all features for improving classification accuracy or decreasing the size of the structure without significantly decreasing classification accuracy of the classifier which is built using only the selected features [22]. Reducing the number of irrelevant/redundant features drastically reduces the running time of a learning algorithm and yields a more general concept. This helps in getting a better insight into the underlying concept of a real-world classification problem [23]. Feature selection methods try to pick a subset of features that are relevant to the target concept and these features are used as the input of classifiers.

The association between the gonioscopic grading and the measured iridocorneal angle features was evaluated for feature selection using Spearman correlation coefficient (*ρ*). There is a high correlation between FD, mAOD, and AT_sl_ and gonioscopic grading as shown in Table 2. We also explored the effect of combining different kinds of features on classifier performance. So, we grouped those features for classification and compared the consistency with other separate features. In the system, these features were firstly normalized to mean zero and variance of one to improve the classification process. Then, the classification was performed based on the normalized features by fuzzy* k*-nearest neighbor classifier (fkNN). The performance of the fkNN was evaluated by comparison with widely used machine learning algorithms, namely, linear discriminant analysis (LDA),* k*-nearest neighbour (kNN), and support vector machines (SVM), to verify the effectiveness of the proposed model.

**Figure 9:**
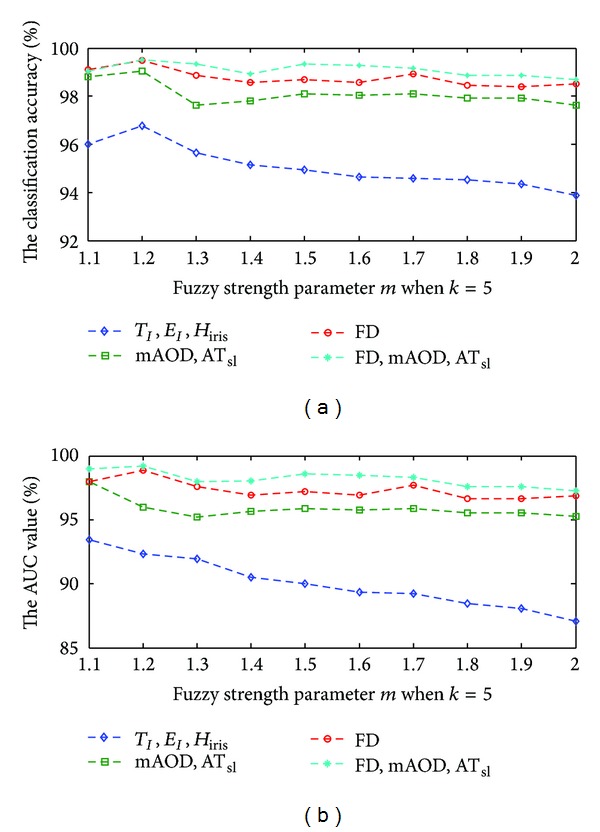
(a) The relationship between the fuzzy strength parameter *m* and the classification accuracy and (b) the relationship between the fuzzy strength parameter *m* and the AUC value.

The classification was performed on the 5-fold cross-validation (5-fold CV) of various combinations of features. To verify the effectiveness of the proposed fkNN classifier, we firstly find the relationship between the classification performance and the fuzzy strength parameter *m* which varies from 1 to 2, with the step size of 0.1. It can be observed that the accuracy was achieved between 93% and 99% and AUC value fluctuates between 87% and 99% based on the feature groups as shown in Figures 9(a) and 9(b). It reveals that fuzzy strength parameter *m* has a big impact on the performance of fkNN classifier. The best classification performance was achieved with the *m* parameter of 1.2.

The average 5-fold CV accuracy and the corresponding standard deviation of fkNN are shown in Table 3. The AUC ranges from 0.88 ± 0.02 to 0.98 ± 0.012. The biometric features and the fractal feature are performed with a similar accuracy. The classification using FD, mAOD, and AT_sl_ features group yields the best performance accuracy 99.11% ± 0.76% and AUC 0.98 ± 0.012. These findings suggest that the proposed method is highly promising in providing accurate diagnosis tools for ACG and will make a greater clinical impact if the study can be done on a larger image database.

We also evaluated the performance of fuzzy kNN by using only three features (FD, mAOD, and AT_sl_) with other classification methods (LDA, kNN, and SVM). All classification algorithms provided a correct classification rate higher than 81%. SVM slightly outperforms LDA with an accuracy of 83.26% ± 4.74% (87.78% ± 7.92% sensitivity and 68.4% ± 9.29% specificity) and an AUC of 0.78 ± 0.03 was reached. kNN was found to outperform LDA as well as SVM with an accuracy of 85.80% ± 5.16% (90.10% ± 5.77% sensitivity and 71.82% ± 5.41% specificity) and 0.80 ± 0.04 AUC value. Minimum differences can be appreciated among LDA, kNN, and SVM classifiers. The best classification performance was provided by the fuzzy kNN classifier with fuzzy membership of the samples (~18%) better than other classifers (LDA, kNN, and SVM) as in Table 4. This is an indication of how important the fuzziness of the membership function is. Additionally, fkNN has been shown to be an efficient and robust classification method to diagnose angle closure glaucoma in SS-OCT images.

The proposed fractal shape descriptor also showed better accuracy in comparison to the moment shape descriptor method. The moment shape descriptor method is shown in the literature as an efficient technique to obtain shape descriptors. However, the results presented in this work suggest that the fractal analysis is a worthy option for providing shape descriptors for classification tasks, as it is invariant to rotation, translation, and scale. Besides, our fractal dimension analysis is performed for each pixel on gray level images that can capture more detailed information of the angle structure. In addition, that fractal shape analysis gives more freedom in computation and is less sensitive to the accurate detection of the landmarks—the scleral spur (SS) and Schwalbe's line (SL).

Moreover, our classification results are comparable to those of Xu et al. [24]. They achieved the classification accuracies of 0.921 ± 0.036 AUC and 84.0% ± 5.7% balanced accuracy at an 85% specificity using histogram equalized pixel (HEP) values and SVM classification in OCT images. We also observed a significant advantage in terms of classification performance of using the fuzzy kNN algorithms in glaucoma diagnosis.

In summary, the proposed framework has been shown to be a useful tool in screening for ACG. While our system can provide useful classification and support to the medical experts through identification of features, human intervention to exploit the extracted knowledge is strongly recommended. We believe that our findings in this study can serve as a basic grading system for angle closure glaucoma diagnosis in the future.

## 4. Conclusions

We proposed a novel automated angle classification system using SS-OCT images. We evaluated several techniques for extracting useful information from SS-OCT images such as traditional biometric parameter measurement and complex shape descriptors using fractal dimension analysis. The experimental results demonstrated that the proposed technique which is the combination of biometric parameter, fractal dimension analysis, and classification by fuzzy kNN method achieved great accuracy in the classification of the open and closed angle glaucoma images with an accuracy rate of 99.11% ± 0.76% and 0.98 ± 0.012 AUC value. The performance of the fully automatic system presented here is comparable to medical experts in detecting glaucomatous eyes and could argue clinicians' diagnosis of angle closure glaucoma.

## Figures and Tables

**Figure 10:**
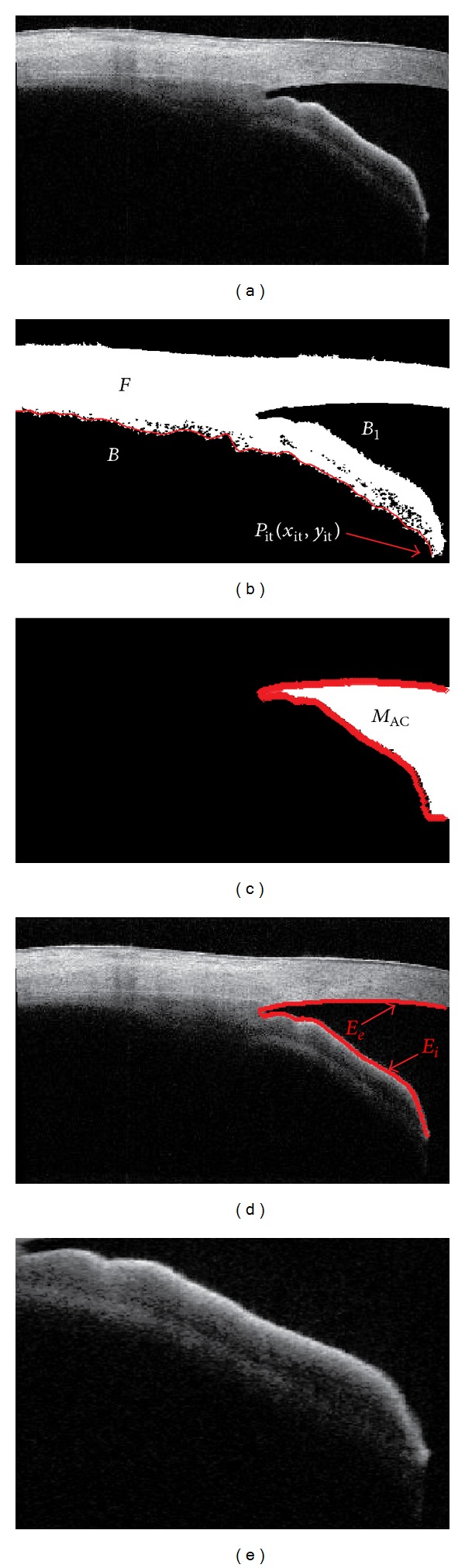
(a) Original SS-OCT image; (b) segmented binary image; (c) segmented anterior chamber; (d) extracted boundaries of lower cornea and upper iris; (e) extracted iris image.

**Algorithm 1:**
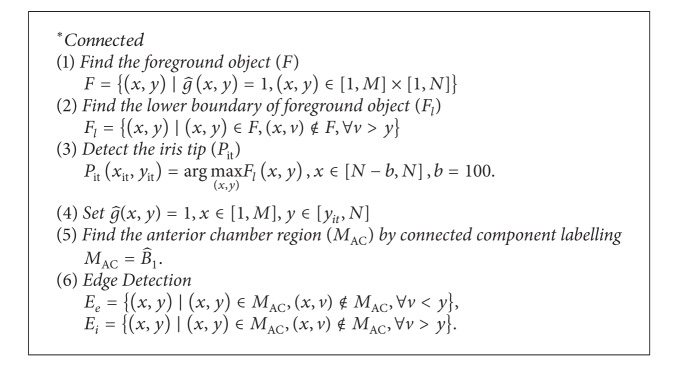
Segmentation of anterior chamber and edge detection.

**Table 1:** Comparison of iridocorneal angle features between open angle and angle closure SS-OCT images.

Features	Open angle images (*n* = 129)		Closed angle images (*n* = 135)	
	Mean (SD)	COV	Mean (SD)	COV
FD	1.87 (0.04)	0.021	1.84 (0.06)	0.03
mAOD	0.35 (0.13)	0.371	0.21 (0.11)	0.523
AT_sl_	0.38 (0.15)	0.395	0.18 (0.04)	0.222
T_*I*_	0.89 (0.09)	0.101	0.85 (0.16)	0.188
*E*_*I*_	0.15 (0.03)	0.2	0.17 (0.056)	0.329
H_iris_	−0.03 (0.04)	1.333	−0.018 (0.039)	2.16

SD: standard deviation, FD: fractal dimension, mAOD: mean of continuous AOD, AT_sl_: area of the trapezoidal profile of iridocorneal angle, T_*I*_: triangularity,  *E*_*I*_: ellipticity, H_iris_: mean curvature of iris, and COV: coefficient of variance.

**Table 2:** The association between the gonioscopic grading and the measured iridocorneal angle features.

	Gonio	FD	mAOD	AT_sl_	T_*I*_	*E*_*I*_	H_iris_
Gonio	1						
FD	**0**.**517**	1					
mAOD	**0**.**582**	**0**.**843**	1				
AT_sl_	**0**.**53**	**0**.**84**	**0**.**979**				
T_*I*_	0.152	0.185	0.193	0.277	1		
*E*_*I*_	−0.16	−0.21	−0.212	−0.273	−0.254	1	
H_iris_	−0.09	−0.021	−0.013	−0.088	−0.065	−0.064	1

Gonio: gonioscopic Shaffer grading.

**Table 3:** The average classification accuracy and the standard deviation of fuzzy kNN classifier with various feature groups (*m* = 1.2,  *k* = 5).

Features	Accuracy (%)		Error rate (%)		Sensitivity (%)		Specificity (%)		AUC	
	Mean	SD	Mean	SD	Mean	SD	Mean	SD	Mean	SD
T_*I*_, *E*_*I*_, H_iris_	94.69	0.71	5.24	0.71	99.9	0.24	77.71	3.39	0.88	0.02
mAOD, AT_sl_	98.21	0.82	1.79	0.82	1.00	0.00	92.14	0.38	0.96	0.01
FD	98.93	0.62	1.07	0.62	1.00	0.00	95.43	2.69	0.98	0.013
FD, mAOD, AT_sl_	99.11	0.76	0.89	0.76	99.9	0.24	96.11	0.24	0.98	0.012

T_*I*_: triangularity, *E*_*I*_: ellipticity, H_iris_: mean curvature of iris, mAOD: mean of continuous AOD, FD: fractal dimension, AT_sl_: area of trapezoidal profile of iridocorneal angle, AUC: area under the curve, and SD: standard deviation.

**Table 4:** Accuracy of fuzzy kNN versus the accuracy of common classification models.

Classifier	Accuracy (%)		Error rate (%)		Sensitivity (%)		Specificity (%)		AUC	
	Mean	SD	Mean	SD	Mean	SD	Mean	SD	Mean	SD
LDA	81.09	4.55	18.90	4.55	80.94	4.84	81.59	8.16	0.812	0.05
kNN (*k* = 5)	85.80	5.16	14.19	5.16	90.10	5.77	71.82	5.41	0.80	0.04
SVM	83.26	4.74	16.73	4.74	87.78	7.92	68.40	9.29	0.78	0.03
fkNN (*k* = 5)	99.11	0.76	0.89	0.76	99.99	0.24	96.11	0.24	0.98	0.012

## Appendices

These appendices deal with the mathematical formulas and explanations of the methodology of SS-OCT images analysis for angle closure detection. We include here Appendices A, B, C, and D for the SS-OCT image preprocessing, segmentation of anterior chamber, feature extraction from SS-OCT images, and classification of open and closed angle detection, which are important parts of the analysis stream for ACG detection.

### A. Preprocessing of SS-OCT Images

We detected the saturation artifacts by searching for abrupt changes in the average intensity of each column of original image *I*(*x*, *y*). The average intensity of each column is defined by

(A.1) $$\begin{array}{c} \mu \left(y\right)=\frac{1}{N}\overset{N}{\underset{y=1}{\sum }}I\left(x,y\right) \end{array}$$

which are compared with the average intensity of the image given by

(A.2) $$\begin{array}{c} \text{avg}\left(I\right)=\frac{1}{MN}\overset{M}{\underset{x=1}{\sum }}\overset{N}{\underset{y=1}{\sum }}I\left(x,y\right). \end{array}$$

Therefore, we can define the artifact region by

(A.3) $$\begin{array}{c} A=\left\{\left(x,y\right)\left|\mu \left(y\right)-\text{avg}\left(I\right)\right|>T,y\in \left[1,N\right],x\in \left[1,M\right]\right\}, \end{array}$$

where *T* is the threshold value and is set to 15. Then, the artifacts are removed by using

(A.4) $$\begin{array}{c} \hat{I}\left(x,y\right)=\{\begin{array}{cc} I\left(x,y\right)-\mu \left(y\right) & \text{if}\;\;\left(x,y\right)\in A,\\ I\left(x,y\right) & \text{otherwise}. \end{array} \end{array}$$

### B. Segmentation of Anterior Chamber and Iris

The anterior chamber region, the lower cornea boundary, and the upper iris boundary are extracted by image segmentation method. First, we convert the artifact removal SS-OCT image $\hat{I}(x,y)$ of dimension *M* × *N* to a binary image defined by

(B.1) $$\begin{array}{c} \hat{g}\left(x,y\right)=\{\begin{array}{cc} 1 & \text{if}\;\;\hat{I}\left(x,y\right)>\text{threshold},\\ 0 & \text{otherwise}, \end{array} \end{array}$$

where the threshold can be calculated by Otsu's method [25], in which the intraclass variance is minimized and the interclass variance is maximized. That binary image is composed of two sets of pixels, the foreground object (*F*) and the background object (*B*). The cornea and the iris can be separated by the connected component labeling [26] segmentation method. The basic idea of this method is to scan the image and group its pixels into components based on connectivity and assign each component a unique label. The segmentation and edge detection algorithm of HD-OCT images depends on whether the cornea is connected or disjointed from the iris as in Tian et al. [9].

The automatic segmentation algorithm of anterior chamber (*M*_AC⁡_) for connected SS-OCT image is shown in Algorithm 1. The segmentation of disjoint SS-OCT images was performed as in Tian et al. [9]. For connected SS-OCT images as shown in Figure 10(a), the background component between the cornea and iris is identified as the anterior chamber (*M*_AC⁡_) from the binary image $\hat{g}(x,y)$ by excluding the background component higher than the iris tip point *p*_it_(*x*_it_, *y*_it_) along the *y*-axis as shown in Figure 10(b). The iris tip is the point which is the maximum value in the lower boundary of the foreground object (*F*_*l*_) as seen in Figure 10(c). The segmented anterior chamber of *M*_AC⁡_ is illustrated in Figure 10(c). Then, the edge of the upper boundary of the *M*_AC⁡_ is denoted by *E*_*e*_ and lower boundary of the *M*_AC⁡_ is denoted by *E*_*i*_ which are extracted as shown in Figure 10(d).

Then, the location of Schwalbe's line which is the point at which there is a maximum distance between the points on the cornea and the regression line is automatically detected as in [9]. The iris image denoted by *I*_*I*_(*x*, *y*) is also extracted from the image *I*(*x*, *y*) by using the following equation:

(B.2) II(x,y)={(x,y) ∣ I(x,y),(x,y)∈[Ei(1,1),M]×[Ei(1,2)−10,N]}.

It is just cropping the original image from the start point to the end point of the inner iris, *E*_*i*_, in the horizontal axis and from the 10 pixels before the start point of the inner iris to the end of the SS-OCT image in the vertical axis to calculate the iris curvature as shown in Figure 10(e).

### C. Feature Extraction of SS-OCT Images

This section describes the analysis of ACA for the extraction of six features by the biometric parameter measurement and shape analysis.

#### C.1. Measurement of Biometric Parameters

The mean iris curvature *H*_iris_ measured from the whole iris image can be quantified as follows.

Let *I*_*I*_(*x*, *y*) be the iris image, where (*x*, *y*)∈[1, *m*]×[1, *n*] are the pixels coordinates and *m* × *n* is the size of the iris image; the curvature denoted by *k*(*x*, *y*) of the iris image is defined in terms of derivatives as

(C.1) $$\begin{array}{c} k\left(x,y\right)=-\frac{I_{Ixx}I_{Iy}^{2}+I_{Iyy}I_{Ix}^{2}-2I_{Ixy}I_{Ix}I_{Iy}}{{\left(I_{Ix}^{2}+I_{Iy}^{2}\right)}^{3/2}}, \end{array}$$

where, *I*_*Ix*_ = *dI*_*I*_/*dx*, *I*_*Iy*_ = *dI*_*I*_/*dy*, *I*_*Ix**x*_ = *d* ^2^ *I*_*I*_/*dx* ^2^, and *I*_*Iy**y*_ = *d* ^2^ *I*_*I*_/*dy* ^2^ are the derivatives in the *x*- and *y*-direction of the iris image. Then, the mean curvature of the iris (*H*_iris_) image is calculated by averaging the principle curvature as follows:

(C.2) $$\begin{array}{c} H_{\text{iris}}=\frac{1}{mn}\overset{m}{\underset{x=1}{\sum }}\overset{n}{\underset{y=1}{\sum }}k\left(x,y\right). \end{array}$$

#### C.2. Anterior Chamber Shape Analysis

The following subsections briefly explain the shape descriptors.

##### C.2.1. Fractal Complex Shape Descriptor (FD)

The fractal dimension for 2D gray level images quantified by differential box counting method (DBCM) was proposed by Sankar and Thomas [27]. The following section reviews the DBC method of FD estimation.

For each pixel (*x*, *y*) in the *I*_IRA_(*x*, *y*) image, we compute the fractal dimension of a small window *W* surrounding the pixel (*x*, *y*). We assigned this fractal dimension to that pixel. We divide *W* into (1/*r*)^2^. DBC presumes that the image belongs to a 3D space *z* = *I*_IRA_(*x*, *y*), where (*x*, *y*) denotes the two-dimensional positions and *z* denotes the gray level of the image. The image with size $\bar{M}\times \bar{N}$ was scaled down by boxes with size *r* × *r* × *h* to many overlapping grids. If the total number of gray levels is *G*, then

(C.3) $$\begin{array}{c} \frac{G}{h}=\frac{\bar{M}}{r}, \end{array}$$

where *r* refers to the compressibility factor, *h* indicates the height of the box, and *G* denotes the range of the gray level in one grid. Supposing that the maximum and the minimum gray values of the image in pixel (*x*, *y*)th grid fall in the box numbers *l*th and *k*th box, respectively, *n*_*r*_(*x*, *y*) = *l* − *k* + 1 denotes the number of needed boxes to cover the grid. Taking contribution from all grids, we have

(C.4) $$\begin{array}{c} {\bar{N}}_{r}=\sum n_{r}\left(x,y\right). \end{array}$$

The value of ${\bar{N}}_{r}$ is determined by the different values of box sizes *r*. After selecting different values of *r* and finding ${\bar{N}}_{r}$, the fractal dimension FD can be obtained by the following equation:

(C.5) $$\begin{array}{c} \text{FD}=\frac{\log\left({\bar{N}}_{r}\right)}{\log\left(1/r\right)}. \end{array}$$

##### C.2.2. Moment Shape Descriptors (*T*_*I*_ and *E*_*I*_)

The triangularity (*T*_*I*_) and ellipticity (*E*_*I*_) moment shape descriptors [17] are computed as follows:

(1) The moment of the iridocorneal angle image denoted by *I*_IRA_(*x*, *y*) can be computed by
  (C.6) $$\begin{array}{c} m_{pq}=\int \int_{I}x^{p}y^{q}dx\;dy,\;p,q=0,1,2,\ldots . \end{array}$$
(2) The central moments can be computed by the following equation:
  (C.7) μpq=∫∫I(x−x¯)p(y−y¯)qdx dy,p,q=0,1,2,…,
  where $\bar{x}=m_{10}/m_{00}$, $\bar{y}=m_{01}/m_{00}$.
(3) Compute the simplest invariants *I*_1_
  (C.8) $$\begin{array}{c} I_{1}=\frac{\mu_{20}\mu_{02}-\mu_{11}^{2}}{\mu_{11}^{4}}. \end{array}$$
(4) The triangularity and the ellipticity can be computed by
  (C.9) $$\begin{array}{c} T_{I}=\{\begin{array}{cc} 108I_{1} & \text{if}\;\;I_{1}<\frac{1}{108},\\ \frac{1}{108I_{1}} & \text{otherwise}, \end{array}\\ E_{I}=\{\begin{array}{cc} 16\pi^{2}I_{1} & \text{if}\;\;I_{1}<\frac{1}{16\pi^{2}},\\ \frac{1}{16\pi^{2}I_{1}} & \text{otherwise}. \end{array} \end{array}$$

### D. Classification

Let *v*_1_, *v*_2_, *v*_3_,…, *v*_*n*_ be the training set of *n* sample vectors and let *V* be a new vector considered as the input to be classified. For a fixed value of *k*, the first step consists of identifying, among these sample vectors, the *k*-nearest neighbours *Y*_1_, *Y*_2_,…, *Y*_*k*_ of the input *V*. Then, the membership vectors of the selected labeled samples *Y* are combined to find the membership vector of the input *V*, where the membership vector describes the probabilities of the membership to the possible *c* classes. Let *u*_*i*_(*V*) be the membership of the input *V* to the *i*th class (with *i* ≤ *c*) and let *u*_*ij*_ be the membership of its *j*th neighbour *Y*_*j*_ to the same class (*u*_*ij*_ = *u*_*i*_(*Y*_*j*_)); the assigned memberships are given as follows (with *m* ≥ 1):

(D.1) $$\begin{array}{c} u_{i}\left(V\right)=\frac{\sum_{j=1}^{k}u_{ij}{\left(1/||V-Y_{j}||\right)}^{2/\left(m-1\right)}}{\sum_{j=1}^{k}{\left(1/||V-Y_{j}||\right)}^{2/\left(m-1\right)}}. \end{array}$$

According to (D.1), the assigned memberships of *V* are influenced by the inverse of the distances from the nearest neighbors and their class memberships *u*_*ij*_. The variable *m* determines how heavily the distance is weighted when calculating each neighbor's contribution to the membership value. If *m* = 2, then the contribution of each neighboring point is weighted by the reciprocal of its distance from the point being classified. As *m* increases, the neighbors are more evenly weighted, and their relative distances from the point being classified have less effect. If *m* = 1, the closer neighbors are weighted far more heavily than those farther away. The ||·|| in (D.1) denotes the Euclidean distance.

The test performance of the classifiers could be determined in terms of classification accuracy, sensitivity, specificity, and area under the receiver operating curve (AUC) as the following equation:

(D.2) $$\begin{array}{c} \text{Accuracy}=\frac{\text{TP}+\text{TN}}{\text{TP}+\text{FP}+\text{FN}+\text{TN}}\times 100\%,\\ \text{Sensitivity}=\frac{\text{TP}}{\text{TP}+\text{FN}}\times 100\%,\\ \text{Specificity}=\frac{\text{TN}}{\text{FP}+\text{TN}}\times 100\%, \end{array}$$

where TP and TN denote the number of true positives and negatives, respectively, and FP and FN denote the number of false positives and negatives, respectively.
